# Personalized Nutrition for Inflammatory Bowel Disease

**DOI:** 10.1093/crocol/otaa042

**Published:** 2020-05-27

**Authors:** Colm B Collins, Helen M Roche

**Affiliations:** 1 UCD School of Biomolecular and Biomedical Science, Conway Institute, University College Dublin, Belfield, Dublin 4, Ireland; 2 Nutrigenomics Research Group, School of Public Health, Physiotherapy and Sports Science, UCD Institute of Food and Health, Diabetes Complications Research Centre, University College Dublin, Dublin, Ireland; 3 Institute for Global Food Security, Queen’s University Belfast, Northern Ireland, UK

## CONCEPTS AND CHALLENGES

Personalized medicine approaches are vital to enhance diagnosis, treatment efficacy, and disease amelioration for inflammatory bowel disease (IBD). Diet, and consequent nutritional status, is a key environmental factor that needs to be included within the larger personalized health paradigm. Thus, the recent focus on personalized nutrition, which attempts to address the diverse nutritional status, nutrient requirements, and potential dietary triggers unique to each patient. While IBD is essentially a chronic inflammatory condition, it would be naive to ignore potential diet-environmental triggers that may prime an aberrant gut immune response. Furthermore, because of the limited efficacy and frequent loss of response of current drug treatments,^1^ pressure is mounting to integrate dietary management into a holistic approach to IBD care. The personalized nutrition paradigm is far better developed for obesity, insulin resistance, and type 2 diabetes risk compared to IBD, despite the close physical inter-relationship between food and the gut. In those areas, dietary intervention studies have been hampered by variability in patient responsiveness due to background diet, different “metabotypes” (or comprehensive metabolic phenotypes), and/or genetic elements.^2–6^ While the mechanistic perspectives have not been fully elucidated, these inconsistent responses pose a significant challenge to understand and advancing dietary therapy. This failure is driving interest in more personalized approaches. To this end, we wish to outline the state of the art and highlight potential for personalized nutrition in IBD.

## NUTRITIONAL STRATEGIES FOR IBD—A BRIEF OVERVIEW OF THE CURRENT STATE OF THE ART

Epidemiologic studies have not identified strong relationships between dietary macro- and micronutrient consumption with incident IBD. The EPIC study demonstrated that no clear dietary pattern was associated with either ulcerative colitis (UC) or Crohn’s disease (CD) risk. It was only when cases occurring within the first 2 years were excluded that a positive association between “high sugar and soft drinks” and “higher UC risk” was found and even then only if they had low vegetable intake.^7^ It is often purported that dairy products, providing calcium, vitamin D, selenium, etc, may be involved in the etiology of IBD by modulating gut microbiota and/or immune responses. However, EPIC also demonstrated only weak association between pre-diagnostic milk consumption and a lower risk of developing CD, in the absence of a clear dose–response relationship.^8^ Pre-diagnostic vitamin D status or vitamin D intake was not associated with IBD risk.^9^ Red meat and processed foods are also commonly perceived triggers of IBD. However, recent interrogation of the Food and Crohn’s Disease Exacerbation Study cohort demonstrated that high (>2 portions per week) or low (<1 portion per month) red and/or processed meat consumption has little impact on the rate of flares in CD.^10^ It should be noted here that this study was directed at identifying triggers of IBD flare rather than triggers of IBD development.

The first systematic review to determine potential relationships between intake of fats, carbohydrates, and protein and subsequent IBD onset suggested an association between increased dietary fats (saturated, monounsaturated, and both n-6 and n-3 polyunsaturated fatty acids), as well as mono- and disaccharides, and IBD risk.^11^ In contrast, low dietary fiber and fruit intake were negatively associated with subsequent CD risk and low vegetable intake was associated with UC. However, most dietary studies were extremely small in terms of subject numbers; therefore, their results should be interpreted with caution. Also given the very different effects of different fats on inflammation,^12^ it is improbable that all fatty acids have equivalent effects in IBD.

Interestingly, a number of studies on concordance rates for monozygotic twins demonstrate a significant increase in incidence among CD and to a lesser extent UC.^13, 14^ However, the relatively low levels of concordance support a strong role for environmental triggers in conferring disease over genetic drivers. Preclinical studies support the concept that early life nutrition impacts chronic colitis susceptibility in later life.^15^ It is unclear if this extends in utero, such that maternal diet during pregnancy may drive IBD susceptibility. However, in a study of a German twin cohort,^14^ firstborn twins had a higher likelihood of developing IBD compared with their trailing sibling. This coincides with greater body mass index among firstborn twins^16^ due to their more optimal position for nutrient intake.^17^ While numerous confounding factors may account for this difference, one possibility is that in utero nutrition may indeed drive IBD susceptibility.

A recent Cochrane review on the impact of dietary intervention on the induction and maintenance of remission in IBD^18^ failed to draw any conclusive findings upon reviewing 18 randomized clinical trials. However, the review was limited to studies of whole food-based dietary studies and ignores the highly effective studies of enteral therapy which will be discussed below. While the review suggests that whole food dietary modification is simply ineffective for the treatment of IBD, this decision should be based upon larger, adequately powered randomized clinical trial in well-defined IBD contexts. The majority of studies have not been adequately powered to detect appropriate disease progression endpoints—thus, valid and robust nutrition science needs to be integrated with clinical medicine in IBD clinical research to advance this field.

## DIETARY INTERVENTIONS: SUCCESSES TO DATE

The heterogeneity of IBD, including discontinuous inflammation which characterizes CD, combined with the zonal nature of intestinal absorption, makes a personalized approach to modifying the patient’s diet all the more critical. Personalized nutritional strategies have potential as either primary and/or adjunct therapies according to different IBD contexts. Exclusive enteral nutrition (EEN) is an effective dietary therapy to induce remission in pediatric CD and is often recommended as a first-line therapy. However, EEN is challenging long term due to poor palatability and recurrence of intestinal inflammation after cessation of EEN.

Elimination diets are very popular, wherein suspected trigger foods are avoided. However, avoiding key dietary elements, such as dairy, red meat, etc., could, in the long term, lead to malnutrition, particularly micronutrient deficiency. A Spanish study suggested that the prevalence of malnutrition was 16% in IBD patients.^19^ However, it should be noted that malnutrition was defined anthropometrically (ie, based upon body mass index, fat free mass) and by subjective global assessments, rather than biochemically, which would provide more detail in relation to actual micronutrient status. The risk of malnutrition is more common in CD rather than UC, and it is determined by extent of the disease activity and duration, reflected in magnitude of the inflammatory response, which in turn can instigate a catabolic and anorexigenic state. Malnutrition can reflect any combination of anorexia with poor food intake, increased nutrient requirements, and increased gastrointestinal losses of nutrients during the active disease state.^20^

Even seemingly well-nourished IBD patients with normal body weight and in remission, are deficient in key micronutrients including selenium, magnesium, vitamin B12, and ferritin, associated with reduced intake of fruits, vegetables, fish, and milk.^21^ A recent work that focused on patient-directed indiscriminate food elimination, describing calcium, vitamin A, and zinc deficiency due to the exclusion of dairy, fish, etc., highlights the need for evidence-based nutritional biochemical profiling, to provide better nutrition education within a comprehensive clinical management plan.^22^

Given the inflammatory nature of IBD, it is understandable that nutritional studies focus on substituting pro-inflammatory with anti-inflammatory nutrients. Indeed, we have demonstrated that dietary fat quantity and quality can differentially affect IL-1β inflammation. Diets rich in saturated fatty acids potentiates NLRP3 inflammasome-mediated IL-1β inflammation,^23^ but monounsaturated fatty acids do not. Other groups have provided very interesting data wherein a range of anti-inflammatory nutrients, including long chain n-3 polyunsaturated fatty acids, curcumin, vitamin D, etc. and/or pre- and probiotics, can downregulate different elements of immunometabolism. Nevertheless, it is very difficult to illustrate these clear and consistent relationships in man.

In a small study by Konijeti et al., the Autoimmune Protocol Diet, used as an adjunct to current therapy, led to clinical remission in 73% of IBD patients in as little as 6 weeks, indicating that more large-scale studies on dietary intervention are certainly warranted. This diet is a modified paleo diet, high in fiber that strives to eliminate potential inflammatory dietary antigens and adjuvants while also preventing dysbiosis. Importantly, this clinical improvement correlated with altered intestinal RNA expression consistent with a cellular response to dietary intervention.^24, 25^

## PERSPECTIVES FOR THE FUTURE—PERSONALIZED NUTRITION VERSUS THE MICROBIOME: HYPE OR HOPE?

Environmental factors play an important role in the pathogenesis of IBD, and diet is a promising and potentially modifiable risk factor for disease onset and severity. It is possible that certain adverse dietary agents may directly or indirectly (via the microbiome) accentuate intestinal inflammation. Conversely, dietary elements may act either in an anti-inflammatory mode or indeed promote the resolution of inflammation. According to the personalized nutrition paradigm, we would purport that one cannot assume that the same dietary insults will trigger IBD onset or resolution in all patients, particularly given the dynamic heterogenous pathology of IBD.

A wealth of research has explored the role of the gut microbiome in IBD—this makes sense given the physical and biological interaction between the gut microbiome and their metabolites with the host gut mucosa. Patients with IBD often have narrowed microbial diversity and altered composition and function of the gut microbiome. While the importance of the microbiome in the IBD field cannot be underestimated dietary diversity may be a key driver of this altered microbiome diversity and functionality? Indeed, if the dietary environment dimension could be incorporated and elaborated within such elegant studies as we have seen for microbiome research, we would greatly clarify the role of diet and nutritional status versus the microbiome in IBD pathogenesis.

Nutrition that potentiates existing IBD therapies represents a largely untapped area for future investigation. Pilot studies using the calorie restriction mimetic, metformin, which greatly re-configures metabolism, demonstrated enhanced responsiveness to malignant melanoma immunotherapy, though these failed to reach statistical significance, likely due to the small sample size.^26^ With the recognition that diet can impact responsiveness to immunotherapy, the question becomes not simply which diet can attenuate intestinal inflammation but also which dietary intervention(s) best potentiates existing IBD immunotherapy in specific IBD patient subcohorts.

Additionally, novel computational methods have led to advances in predicting nutritional treatment approaches for diabetes. Machine learning algorithms that integrate data on blood parameters, diet intake, anthropometrics, and gut microbiota can accurately predict personalized postprandial glycemic response to meals.^27^ Adapting a similar machine learning approach to develop IBD appropriate algorithms is necessary to integrate such a complex system as human nutrition into the clinical management of IBD.

In conclusion, while there is insufficient evidence to demonstrate a clear link between specific dietary factors and IBD pathogenesis, a number of things are clear. Firstly, the contribution of diet should equal those of genetics, immune function, and the microbiome in the classic IBD pathogenesis Venn diagram. Secondly, an individualized, highly powered and comprehensive approach is needed to understand the complex contribution of diet to intestinal inflammation, disease relapse, and optimization of IBD medication.
